# Frey’s Syndrome: A Review of Aetiology and Treatment

**DOI:** 10.7759/cureus.20107

**Published:** 2021-12-02

**Authors:** Angelos Mantelakis, George Lafford, Chang Woo Lee, Harry Spencer, Jean-Luc Deval, Anil Joshi

**Affiliations:** 1 Plastic and Reconstructive Surgery, Guy’s and St. Thomas’ NHS Foundation Trust, London, GBR; 2 Plastic Surgery, Royal Victoria Infirmary, Newcastle upon Tyne Hospitals NHS Foundation Trust, Newcastle, GBR; 3 Plastic and Reconstructive Surgery, St. George’s University Hospitals NHS Foundation Trust, London, GBR; 4 Plastic and Reconstructive Surgery, Addenbrooke’s Hospital, Cambridge University Hospitals NHS Foundation Trust, Cambridge, GBR; 5 Otolaryngology - Head and Neck Surgery, Lewisham and Greenwich NHS Trust, London, GBR

**Keywords:** parotid gland injury, facial surgery, parotid surgery, gustatory sweating, total parotidectomy, frey's syndrome

## Abstract

First described by Polish Neurologist Łucja Frey in 1923, Frey’s syndrome (FS), or auriculotemporal syndrome, is a rare condition characterised by gustatory sweating, typically encountered as sequelae following invasive head and neck surgery. The pathophysiology of FS can be described by aberrant reinnervation of postganglionic parasympathetic neurons to the surrounding denervated sweat glands and cutaneous blood vessels.

Multiple invasive procedures have been associated with FS ranging from salivary gland surgery to burn reconstruction and thoracoscopic sympathectomies. Rarely, FS can be secondary to trauma or non-surgical aetiologies, including diabetes and infection.

Physical symptoms vary based on the severity and surface area affected by FS and range from mild symptoms to severe psychosocial morbidity for patients. Surgeons operating in the head and neck, including otolaryngologists, maxillofacial surgeons, and plastic surgeons, should be aware of this potential complication and be up to date with diagnosis and treatment strategies for FS.

This review article summarises the literature relating to FS focusing on its aetiologies, symptomatology, prevention, and available treatments, aiming to provide an up-to-date review of this condition for surgeons operating in the head and neck region. Although various treatment options have been suggested, these are often limited to topical agents that require life-long administration for symptom control. Further research is recommended to identify the optimal treatment for this condition and the role of surgery as a treatment for severe or refractory cases.

## Introduction and background

Frey’s syndrome (FS) or gustatory sweating was first described by Polish neurologist Łucja Frey in 1923 [1]. This disorder is characterised by unilateral sweating and flushing of the facial skin that occurs during meals, usually in the area of the parotid gland [2]. It is a well-described pathology resulting from damage to the auriculotemporal nerve which contains both sympathetic and parasympathetic nerve fibres, respectively, decreasing and increasing saliva production in the parotid. Damage may occur during parotidectomy or from facial trauma as well as other rarer aetiologies, including forceps delivery, thoracic sympathectomy, and burns injuries [3]. The disrupted parasympathetic secretomotor fibres normally destined for the parotid are misdirected during regeneration. Instead, these fibres aberrantly reinnervate sweat glands and cutaneous vessels in the territories of the auriculotemporal and large auricular nerve distributions [4]. Thus, a gustatory stimulus may give rise to sweating, flushing, and heating in the regions anterior to the ear, over the angle of the mandible, and over the site of the parotid [5]. Although these symptoms often vary in frequency and severity among patients, they generally tend to be exacerbated during mastication and by foods producing a strong salivary response.

Symptoms of FS present when the injured nerve is regenerated, which may vary from a few weeks to several months after the initial insult [6]. A diagnosis can be made clinically or confirmed objectively with the use of the starch-iodine Minor test or infrared thermography [7]. While physical symptoms are generally considered mild, FS can be a major source of psychosocial morbidity for patients [3]. Although a minority of patients suffer from severe symptoms, quality of life assessments of patients undergoing parotid surgery have identified FS as a significant self-perceived sequela [7]. This may be further compounded by the fact that patients report associated discomfort that worsens for up to two years after the initial onset [8,9]. Current management options for patients with FS are primarily medical, with surgical techniques reserved for those with symptoms refractory to conservative measures. As such, this article aims to provide a review of the causes and treatment options for this condition.

## Review

Salivary gland surgery

Parotidectomy

Parotid tumours account for roughly 3% of head and neck neoplasms, of which 70-80% are benign [10]. Major surgical options for benign parotid tumours (BPTs) are extracapsular dissection (ECD), partial superficial parotidectomy (PSP), superficial parotidectomy (SP) and total parotidectomy, with PSP emerging as the most commonly utilised approach in recent years [11-13]. The incidence of FS post-parotidectomy varies greatly in the literature, potentially due to the subjective nature of defining the disorder. On objective testing using the Minor test, approximately 100% of patients demonstrate evidence of gustatory sweating post-parotidectomy, raising the question of whether this is an inevitable consequence of the surgery. In comparison, only 10-30% of patients independently report and seek help for symptoms, while a further 20-60% of patients admit to having symptoms of FS on questioning [3,14]. Despite this, auricular-temporal FS is a well-established and prevalent complication of parotid gland surgery, which represents the most frequent cause of the condition [5].

Analysis of patient factors has revealed that the only statistically significant clinical predictor of developing FS post-parotidectomy is tumour size (p = 0.018). The incidence of FS approximately doubles in patients with tumour size of 4 cm or greater, relative to those with tumour size less than 4 cm [15]. Factors such as age, sex, tumour location, disease pathology, and previous radiotherapy do not appear to be associated with the development of the condition [15,16]. In benign disease, studies have failed to identify any single risk factor for the development of FS [17]. Given its relatively high incidence and paucity of specific predictors, there has been great interest in identifying preventative measures against the development of FS. Meta-analyses of various types of parotidectomies have reported that PSP is associated with a lower prevalence of FS than SP (odds ratio [OR] = 0.54 (0.33-0.9); p = 0.02), while the prevalence of FS after ECD is lower than PSP (OR = 0.12 (0.03-0.48); p = 0.003). There are no significant increases in the rates of tumour recurrence between PSP and SP or ECD and PSP [10,18].

FS is a recognised complication following neck dissection. A retrospective study at the University of Vienna found that eight out of 395 patients (2.0%) who underwent ECD for BPTs went on to develop FS [19]. The same study also found that the incidence of FS following SP was lower (1.8%) than that of ECD. Despite this, a recent large-scale systematic review and meta-analysis that investigated complications associated with ECD versus SP for parotid tumours found the opposite to be true. The study included a total of 1,641 patients from seven studies and investigated the occurrence of FS as well as other complications such as facial nerve injury, infection, and salivary fistula/sialocele [20]. The review found that only 0.7% of patients who underwent ECD went on to develop FS while the incidence of FS among patients who underwent SP was 10.0% [20]. It appears that the incidence of FS occurring after neck dissection is lower than the incidence following parotidectomy, although some studies have demonstrated a higher incidence of FS following neck dissection. This can be attributed to surgical technique and extent of neck dissection, although it is currently unclear and further research is needed to explain these differences.

Submandibular Gland Surgery

While there is a robust association between parotid surgery and auricular-temporal FS, the link between submandibular surgery and FS is more tenuous. A 2012 review identified 16 cases in the literature that reported gustatory sweating following submandibular gland excision [20]. Compared to the relatively common development of FS after parotidectomy, another study reported only 1% (1/68 cases) of patients developing gustatory sweating after submandibular extirpation [21]. The pathophysiology is thought to be similar to that of auricular-temporal FS. The chorda tympani carries taste fibres from the anterior two-thirds of the tongue, along with vasodilatory and parasympathetic secretomotor fibres, to the submandibular glands. Aberrant nerve regeneration after sectioning of these fibres while accessing the submandibular gland is thought to give rise to this gustatory sweating syndrome [22]. Lingual nerve blocks have been used to confirm the involvement of the chorda tympani, and the nomenclature chorda tympani FS has been used to distinguish it from the aforementioned auricular-temporal FS [20].

The disparity of the incidences between auricular-temporal and chorda tympani FS is poorly understood but may be multifactorial. Parotidectomy is a significantly more common procedure than submandibular gland excision, and therefore, absolute numbers may account for a higher rate of FS [21]. It has also been hypothesized that the relatively deep location of the lingual nerve may account for the rarity of chorda tympani FS. Regenerated nerves must travel further to reinnervate superficial sweat glands from the chorda tympani than from the secretomotor fibres of the auricular-temporal nerve. This increased distance and the associated tissue barriers may reduce the likelihood of anastomotic communication [22]. Despite this, the use of interposition grafts to prevent chorda tympani syndrome has been cited in the literature, while other recommendations include using a minimally invasive technique to approach the submandibular gland and treating the lingual nerve and submandibular ganglion securely [20,22].

Head and neck surgery: Other

Facelifts

Facelifts and rhytidectomy are further surgical procedures that may be associated with FS [23]. During rhytidectomy, it is possible to cause iatrogenic damage to the underlying structure in the parotid region [24]. Despite this, there are currently no published cases of FS developing after cosmetic facial surgery. The facelift incision and the lazy-S (Blair’s incision) incision are two surgical techniques commonly used for parotidectomy. A recent retrospective analysis compared the incidence of FS following parotidectomy using a modified version of the facelift incision with the traditional lazy-S incision. They found that there was a significantly lower incidence of FS when the modified facelift incision was used [25]. Although this was a small study that only investigated 61 patients, the results suggested that surgical technique influences the incidence of FS. As these techniques are commonly involved in rhytidectomy and facelifts, the risk of developing FS after these procedures may be influenced by the surgical approach but more evidence including randomised control trials and retrospective analysis is needed to further assess this possibility.

Carotid Endarterectomy

Another possible but rare cause of FS following head and neck surgery is iatrogenic injury following carotid endarterectomy. This method of injury was first reported in 1999 but there have been no such cases published on Medline since [26]. Given that carotid endarterectomy is a relatively common procedure, the lack of reported cases would suggest that FS remains a rare complication of carotid endarterectomy. First bite syndrome has also been recognised as a complication of carotid endarterectomy, which is a condition thought to arise from damage to the sympathetic nerves supplying the parotid gland, similar to the suggested pathophysiology of FS [27-30].

Thyroidectomy

FS is not a known complication following total thyroidectomy. However, there is a single case report in which the patient developed a sudden, transient episode of unilateral facial flushing in the recovery room [28]. This was a single, transient, and self-resolved episode of FS post-elective thyroidectomy likely secondary to irritation of the cervical portion of the truncus sympathicus following the operation.

Trauma

Mandibular Fractures/Dislocations

FS is also a recognised complication of trauma to the head and neck. This is a rare event that has been scarcely reported in the literature. One recognised pattern of injury associated with the development of FS is fracture or dislocation of the mandibular condyles in the region of the parotid gland. Cases of FS following fracture of the mandible have been documented as far back as 1969 [30]; however, FS remains a rare complication of mandibular fracture or dislocation, and therefore, very few cases have been reported [31]. A systematic review analysed ten case reports and one prospective study investigating the occurrence of FS after closed treatment of mandibular condyle fractures. From the ten case reports, it was found that the mandibular condyle was dislocated in six cases and only one of the ten cases showed a spontaneous remission of symptoms [31]. The prospective study reported two cases of FS in 237 fractures of the mandibular condyle managed by closed treatment over a five-year period which represents an incidence of approximately 0.8% [32]. Since this study, there has also been a case of congenital FS occurring in a patient with bilateral trifid mandibular condyles, although this is currently the only report of such a case [33]. The mandible is the third most commonly fractured facial bone with most cases of mandibular fracture occurring as a direct result of trauma [34]. The small number of reports of FS following fracture of the mandible can be because the condition is underreported, but with the current paucity of evidence surrounding mandibular fractures and FS, it is currently not possible to accurately estimate the incidence of the condition.

Penetrating Injuries

Penetrating trauma to the parotid region is often described throughout the literature as a possible cause of FS [35,36]. Łucja Frey’s initial account of auriculotemporal nerve syndrome in 1923 described a man who had been shot in the left mandible with a rifle bullet [37]. The patient went on to develop a fistula in the internal auditory meatus, and following a further incision in the place of the first wound developed symptoms of redness, warmth, and sweating in the parotid area while eating [38]. Despite this, there are very few case reports documenting this type of injury. The earliest case was reported as far back as 1726 following a penetrating wound to the left side of the face by a deer following a hunting accident [39]. With such a small volume of published material on this topic, it is impossible to accurately comment on the incidence of FS following facial trauma, although the lack of documented cases would suggest that it is a rare complication following this type of injury.

Burns

There has been a reported case of FS following a full-thickness burn to the right forehead, cheek, and temporal region of a patient. The tissue was excised and reconstructed with split-thickness skin grafts at the time of injury which may have also contributed to the development of FS [40].

Forceps Delivery

FS presenting during infancy is rare and is often misattributed to food allergy as symptoms often occur around the age that children begin to consume a varied diet [41]. One review found that out of eight cases of FS in infants, six of the cases were associated with forceps delivery [20]. The same review found that out of 28 reported cases of infantile FS in the literature, 14 were associated with forceps delivery, suggesting that the trauma of forceps delivery may be a contributing factor to infantile FS [42].

Thoracoscopic sympathectomy

Thoracoscopic sympathectomy is a surgical procedure involving the removal of part of the thoracic sympathetic nerve trunk. It is often performed in cases of hyperhidrosis where medical management has failed to alleviate symptoms. FS is a well-recognised side effect following thoracic sympathectomy, although the reported incidence of FS occurring following thoracoscopic sympathectomy varies greatly in the literature. One study reported gustatory sweating side effects occurring in as many as 50.7% of cases while other studies have reported the incidence to be as low as 5.5% [43,44]. It has also been suggested that the extent of sympathectomy may also influence the incidence of FS. A large-scale retrospective analysis examined a total of 238 patients who had undergone thoracoscopic sympathectomy to treat hyperhidrosis and found that FS was reported in 32% of patients. They also found that FS occurred more commonly (44%) when sympathectomy was performed in the region of T2-T4 compared to sympathectomy performed at the T2 (27%) and T2-T3 (29%) regions [45]. Another study by Neumayer et al. compared thoracoscopic sympathectomy performed at the level of T2-T4 with a limited sympathectomy performed solely at the level of T4 in the treatment of palmar hyperhidrosis. Incidence of FS, the success of the procedure, and patient satisfaction were all investigated, and it was found that gustatory sweating was markedly reduced in the T4 group (33.3 vs. 2.1%; p = 0.0019) while there was no difference in the resolution of hyperhidrosis between the two groups with both groups maintaining 100% patient satisfaction scores [46]. The results of this study are also supported by a recent meta-analysis which demonstrated a significantly lower occurrence of FS when sympathectomy was performed at the level of T4 when compared with T3 [47]. These results suggest that restricted low-segment thoracic sympathectomy can decrease the incidence of FS. It is worth noting that the same analysis found no significant difference in the incidence of FS when comparing thoracic sympathectomy performed at the level of T2 or T3 [48]. There has also been discussion surrounding a link between the location of the hyperhidrosis being treated and the incidence of FS, although the extent of sympathectomy is determined by the location of primary hyperhidrosis, and therefore, further randomised controlled trials are needed to distinguish between these two factors [45].

Non-surgical causes

Infection

Several sources often cite parotitis and parotid abscesses as a potential cause of FS. Despite this common assumption, literature analysis reveals that there are no reports of FS occurring after an isolated bacterial infection. As such, this association warrants further investigation.

On the other hand, there have been documented cases of FS following herpes zoster infection [48,49]. These patients, a two-year-old girl and a 27-year-old man, were diagnosed and treated for herpes zoster in the distribution of the mandibular nerve. After three and twelve months, respectively, they reported symptoms of gustatory sweating in the regions previously affected by herpes zoster. These symptoms persisted for up to seven years. Both patients were subsequently able to achieve adequate symptomatic control with conservative measures.

Diabetes

For many years, the development of FS in diabetics was documented in the literature only in the form of case reports, suggesting that it was a rare occurrence [50,51]. A paradigm shift occurred when Shaw et al. reported that 36% and 69% of patients with diabetic neuropathy and nephropathy, respectively, suffered from FS compared to less than 5% of patients in the control groups (non-nephropathic/neuropathic diabetics and non-diabetic renal failure) [52]. Factors correlating to increased incidence of FS in diabetes include the presence of autonomic neuropathy and diabetic nephropathy. Sex, diabetes type, diabetes duration, and serum creatinine did not appear to impact the development of FS in diabetics.

The mechanism of development of FS in diabetes is not well understood. Axonal regeneration is known to occur in diabetic neuropathy, and the generally accepted explanation is that regenerating fibres from the vagus follow sympathetic nerves to facial sweat glands [52]. The identification by Shaw et al. of the link between nephropathy and FS complicated the understanding of this mechanism. Possible hypotheses proposed include an autoimmune mechanism, nephropathic metabolic changes, and diminished central control of salivatory reflexes.

Prevention of Frey’s syndrome

Recognising the high incidence of FS after a parotid surgery, attempts have been made to prevent its development intra-operatively. Based on its pathophysiology, two ideas have been pioneered. Kornblut et al. pioneered the idea of placing a barrier between the parotid wound and the overlaying cheek skin to prevent the aberrant parasympathetic nerve regeneration [53]. Since then, allografts, such as acellular dermal matrix, or autografts, such as sternocleidomastoid muscle flap, temporal fascial flap, superficial musculoaponeurotic system flap, platysma muscle flap, and free fat grafting have been described in the literature for this purpose [54]. Another idea was pioneered by Singleton and Cassisi suggesting a thicker skin flap could prevent the development of FS by protecting the sweat glands from being exposed to aberrant parasympathetic nerve regeneration [55].

A meta-analysis of 14 randomised clinical trials involving 1,098 patients showed that the use of an acellular dermis matrix reduced the risk of FS by 82% compared with the no-graft group based on an objective starch-iodine test, and by 90% compared with the no-graft group based on a subjective assessment [17]. The use of a muscle flap reduced the risk of FS by 81% compared with the no-graft group [17]. The meta-analysis also showed that prevention could not be always be achieved with the interposition of grafts intra-operatively [17]. FS developed in 8.3% of patients in the acellular dermis matrix group and 11.1% of patients in the muscle flap group [17]. Furthermore, it is currently difficult to predict which patient will most likely develop FS post-operatively [56]. This poses a challenge to surgeons in deciding to whom they should offer preventative intra-operative measures.

Increased skin flap thickness during parotidectomy may greatly reduce the skin surface area affected by FS post-operatively. Durgut et al. conducted a randomised clinical trial to evaluate the association between skin flap thickness and FS in parotid surgery. A total of 30 patients were randomised into two groups: the subcutaneous group and the sub-superficial musculoaponeurotic (sub-SMAS) skin elevation group. They measured the skin flap thickness with a micrometre at defined points, and the patients were assessed for FS post-operatively using both the subjective and the objective (starch-iodine) assessments. They showed that the thickness of the skin flap in the subcutaneous group was significantly less than the sub-SMAS group, but there was no association between the skin flap thickness and objective FS [57]. However, they demonstrated a decrease in the total skin surface area affected with an increased flap thickness: 0.5 cm^2^ (0-18 cm^2^) in the sub-SMAS group compared to 7.5 cm^2^ (0-48 cm^2^) in the subcutaneous group [3].

Treatment options for Frey’s syndrome

Linder and colleagues’ analysis of their patients (120 patients retrospectively and 23 patients prospectively) showed that of the 31 patients who developed FS (21 from the retrospective analysis and 10 from the prospective analysis at 12 months), 77% (n = 24/31) were reassured by an explanation of the condition alone and wanted no further treatment [3]. However, 23% (n = 7/31) of the patients became socially debilitated from social embarrassment caused by their severe symptoms and asked for further treatment. This study highlighted that FS can be associated with high psychosocial morbidity and implies treatment indication for FS is mainly of patient’s choice.

Various medical and surgical treatment options have been reported in the literature with varying success rates. Currently, there are no agreed management guidelines available for FS. This is because there are no randomised or quasi-randomised controlled trials involving patients diagnosed with FS using a clinical standard such as the starch-iodine test, receiving any intervention, compared to no treatment or an alternative intervention [58].

Medical Management

Topical application of antiperspirant or anticholinergic agents, such as scopolamine, aluminium chloride, and glycopyrrolate, and intracutaneous injection of botulinum toxin type A (BoNT-A) have been described.

The main advantage of topical antiperspirant or anticholinergic application is in its non-invasive nature. Black and Gunn reported the use of topical application of aluminium chloride hexahydrate 20% w/v in an alcoholic solution using a roll-on applicator [59]. They asked their patients to apply the solution to the affected area at night. They demonstrated that the treatment was effective in improving symptoms, as assessed by the starch-iodine test. However, there is no agreed application regime. For example, Linder et al. used the same solution but asked their patients to apply it 30 minutes before eating only [3]. Furthermore, subjective assessment of treatment outcome varied among patients, an immediate symptom recurrence on stopping treatment was noted, and the patients’ long-term compliance was very poor [3,59]. No physical side-effects have been reported allowing the short follow-up period and poor patient compliance [58,59]. With regards to topical anticholinergic application, various preparations (for example, 0.25%, 1%, or 3% scopolamine in either solution or a cream form) with no agreed regimen have been tried with varying success rates [60]. While suffering from the same disadvantages as the topical antiperspirant application, physical side-effects associated with anticholinergics have also been reported (for example, dry mouth) [60].

BoNT-A is a neurotoxin that blocks the presynaptic release of acetylcholine at the neuromuscular junction causing chemodenervation. A recent meta-analysis of 22 published case series concluded that intracutaneous injection of BoNT-A is not only effective in treating FS but also safe with minimal side-effects [61]. Of the 411 patients reviewed in this study (follow-up period of 1-29 months), only two patients had no response. The mean duration of effect of the BoNT-A injection ranged from three to twenty months. In terms of side effects, of the 411 patients, five patients suffered from dry mouth, and eight patients suffered from transient muscular weakness or numbness, which fully resolved in 12 weeks. The main side-effect reported was pain after or at the time of injections by 30 patients. Jansen et al. retrospectively analysed 100 patients treated with BoNT-A injection over 16 years [62]. They found that 70.5% of their patients needed two or more injections. If two or more injections were needed, the average number of treatments needed was 5.8 ± 4.2 (median number of treatments: 4). The median time interval between treatments was 12 months (range: 0.23-125.5 months). Currently, there is no agreed BoNT-A dosage and application regime for FS (61,62). Following their meta-analysis, Xie et al. suggested using BoNT-A concentration of 2.0-5.0 U/mL, with 0.1 mL volume per injection, with the maximal dose no more than 380 U per participant, and the interjection distance varying from 10 to 20 mm [62].

Surgical Management

Surgical management of FS described in the literature broadly falls into two categories: a neurectomy or the placement of a physical barrier between the parotid bed and the overlying skin.

An intratympanic approach to remove the tympanic plexus in the middle ear has been reported based on the understanding that preganglionic parasympathetic fibres to the parotid gland are conveyed by the tympanic plexus. In an analysis of 73 cases, Hays showed that the approach was successful in 56% of cases, with an additional satisfactory result in 26% of cases [60]. Symptom recurrence, albeit less severe, was common, which was thought to be caused by the regrowth of the tympanic plexus [59].

Based on the same concept as the preventative intra-operative technique, interposition of a physical barrier between the parotid wound and the overlying cheek skin has been tried to disrupt the aberrant nerve regeneration and treat FS. An open approach involving re-elevation of the cheek skin flap to place a dermal graft, dermal collagen implants, or a temporoparietal fascia flap has been reported [63-65]. Less invasive fat injection has also been reported avoiding the need to re-elevate the skin flap [66]. Despite the previously reported success of a single-layer barrier technique in preventing symptom recurrence, Dai et al. were not able to achieve good results [67]. They tried the double barrier technique using a combined sternocleidomastoid muscle and temporalis fascia flaps, demonstrating >50% of patients having complete resolution by starch-iodine testing, with all patients reporting a significant reduction in the average surface area of gustatory-sweating-positive skin from 12.80 to 1.32 cm^2^ post-operatively [67]. They further concluded that their technique greatly improved the facial asymmetry secondary to parotidectomy [67].

Surgical management appears feasible. However, given the increased risk for facial nerve injury with either approach, along with other possible surgical complications, surgery should only be considered in severely symptomatic patients refractory to medical therapy.

## Conclusions

Although there are several reported causes of FS in the literature, gustatory sweating remains a well-recognised and established complication of invasive head and neck surgeries. In this review, we have identified a wide range of potential aetiologies that surgeons should be aware of to prevent FS and to appropriately prognosticate their patient’s regarding the risk of gustatory sweating. When deemed at high risk of developing FS, there is evidence to suggest that preventative intra-operative techniques such as muscle flap reconstruction or the use of acellular dermal matrices may reduce both the incidence and the area affected by FS post-operatively. To date, there has been no definitive cure for this condition. Management of FS is primarily medical, and often lifelong, through the utilisation of topical agents or botulinum toxin. However, these mandate continuous administration with reversal of symptoms upon cessation of treatment. Although reported in the literature, there is currently no established role of surgery in the treatment of FS. Evidence for the effectiveness of reported techniques is confined to level 4 and 5 evidence including case reports and short case series. Further research is required to identify the optimal treatment for this condition.
